# Decision-making out of neural events: from discrimination information to psychometric power laws

**DOI:** 10.1186/1471-2202-14-S1-P153

**Published:** 2013-07-08

**Authors:** Javier A Caballero, Nathan Lepora, Kevin N Gurney

**Affiliations:** 1 Department of Psychology, The University of Sheffield, Sheffield, S10 2TN, UK

## 

**Figure 1:**
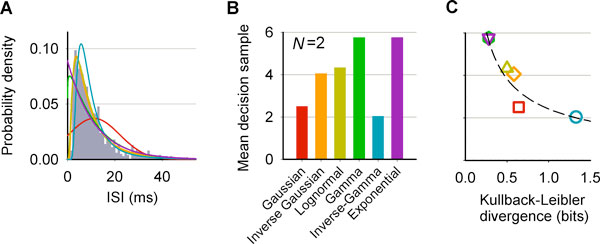
**(A) PDFs of interest fitted to ISIs (grey bars) recorded in **[1]**from the MT during the RDMT**. **(B) **mean decision sample for each MSPRT realisation (accuracy 95% over 1000 trials). **(C) **the values in **(B) **vs the fa to fb KLD. The dashed line is a fitted power law. All colour coding as in panel **(B)**.

The strength/intensity of the stimulus in the random dot motion task (RDMT) [1] is determined by the percentage of dots in the kinematogram moving towards a saccadic target, *a*. Due to the uncertainty in the stimuli, neurons in sensory systems have evolved to transform environmental information, comprising evidence upon which a decision can be made (e.g. saccading to *a*). The neurons in the middle-temporal area (MT) appear to produce such evidence during the RDMT, given their tuning to a 'preferred' direction of visual motion. If the dots move predominantly in the preferred direction of an MT neuron, it generates inter-spike intervals (ISI) supporting a saccade to *a*. These ISIs seem randomly sampled from a distribution, *f_a_*, with mean, *μ_a_*. Otherwise, the ISIs follow another distribution, *f_b_*, with mean, *μ_b_*, where *μ_b _*is larger than *μ_a _*and this difference increases with stimulus strength. The accuracy vs motion-strength function of an ideal observer provided with empirical distributions like *f_a _*and *f_b_*, from a single MT neuron, approximates the subject's psychometric function (at the behavioral level) [1]. The distributions *f_a _*and *f_b _*are non-negative, positively skewed and have a mode larger than 0 (figure 1A), as is typical for neural events recorded in many brain areas. Here we investigate why this is advantageous for decision-making. As theoretical decision-making units, we produced 5 new instantiations of the multi-hypothesis sequential probability ratio test (MSPRT) [2]. Each unit assumes its stream of input evidence to follow 1 of 5 probability density functions (PDF) whose compatibility with the empirical distribution of ISIs varies (figure 1A). These include the Inverse Gaussian, Lognormal, Gamma, Inverse-Gamma and Exponential PDFs (the latter is the distribution of the inter-event times in the oft-used Poisson process). Under equal and appropriate conditions, we then compared their mean decision sample with that of an MSPRT instantiation that assumes Gaussian inputs proposed in [3] and discussed in general in [4], as exemplified in figure 1B. The mean decision sample is the mean number of observations required by a unit to identify which of *N *parallel information sources supports saccading to *a*, with a given accuracy. This decision sample is a model of the 'neural decision time'; the psychophysical reaction time also includes sensory and motor delays. The pattern of our results is explicable using a measure of the discrimination information between *f_a _*and *f_b_*, i.e. the Kullback-Leibler divergence (KLD). We found that, the mean decision sample decreases with increasing *f_a _*to *f_b _*KLD and, crucially, this follows a power law (figure 1C). At the behavioral level, Piéron [5] reported the mean reaction time to the presentation of a stimulus (go/no-go decision-making) being shorter for more intense stimuli, and that a power law relates these measures. The universality of Piéron's law indicates that it can inform us of something fundamental about sensorimotor decision-making. Our results suggest that its explanation could lie in the power law relationship between the mean neural decision time and the discrimination information (KLD) among the distributions of sensory evidence.
